# Responses of attached bacterial communities to blooms of the swimming shelled pteropod *Creseis acicula* in Daya Bay, southern China

**DOI:** 10.1093/femsec/fiae034

**Published:** 2024-03-23

**Authors:** Rongjun Shi, Tingting Han, Zhanhui Qi, Honghui Huang

**Affiliations:** Guangdong Provincial Key Laboratory of Fishery Ecology and Environment, Key Laboratory of Open-Sea Fishery Development, Ministry of Agriculture and Rural Affairs of China, South China Sea Fisheries Research Institute, Chinese Academy of Fishery Science, Guangzhou 510300, China; Guangdong Provincial Key Laboratory of Fishery Ecology and Environment, Key Laboratory of Open-Sea Fishery Development, Ministry of Agriculture and Rural Affairs of China, South China Sea Fisheries Research Institute, Chinese Academy of Fishery Science, Guangzhou 510300, China; Guangdong Provincial Key Laboratory of Fishery Ecology and Environment, Key Laboratory of Open-Sea Fishery Development, Ministry of Agriculture and Rural Affairs of China, South China Sea Fisheries Research Institute, Chinese Academy of Fishery Science, Guangzhou 510300, China; Guangdong Provincial Key Laboratory of Fishery Ecology and Environment, Key Laboratory of Open-Sea Fishery Development, Ministry of Agriculture and Rural Affairs of China, South China Sea Fisheries Research Institute, Chinese Academy of Fishery Science, Guangzhou 510300, China; Southern Marine Science and Engineering Guangdong Laboratory (Guangzhou), Guangzhou 511458, China

**Keywords:** bloom, bloom-sensitive particle-attached bacteria, *Creseis acicula*, free-living bacteria, particle-attached bacteria

## Abstract

The shelled pteropod *Creseis acicula* is a marine pelagic shellfish widely distributed from temperate to tropical seas around the world. From June to July 2020, a *C. acicula* bloom first happened in the Daya Bay, southern China, and its density reached the highest value (5600 ind. m^−3^) ever recorded around the world. However, few studies have investigated the responses of bacterial communities to the *C. acicula* bloom. In the present study, we examined the community profiles of three communities of bacteria including the free-living and particle-attached bacteria in the blooming and reference waters, and bacteria attached to the whole body and shell of *C. acicula* using a high-throughput sequencing method. The results indicated that the *C. acicula* bloom had a greater impact on particle-attached bacteria than free-living bacteria. Among the bloom-sensitive particle-attached bacteria, the predominant bacterial phyla were Pseudomonadota, Bacteroidota and Verrucomicrobiota in the blooming areas, whereas they were Actinomycetota and Planctomycetota in the reference areas. Specifically, fecal bacteria *Haloferula* and *Halioglobus* spp. were significantly enriched in the blooming waters and accumulated on *C. acicula* shells. Conversely, the significantly lower relative abundance of *Nocardioides* sp. in the blooming area and accumulated on the whole body of *C. acicula* indicated their attachment to particles consumed by *C. acicula*. Overall, our results suggested that the *C. acicula* bloom influenced marine bacteria, particularly particle-attached bacteria, by increasing (e.g. providing shells and feces) or decreasing (e.g. filter-feeding the suspended particles) the abundance of available substances.

## Teaser

A bloom of *Creseis acicula* exerted both direct and indirect effects on bacterial communities through filter feeding, respiration, metabolite release, excretion and decomposition, impacting particle-attached bacteria more than free-living bacteria.

## Introduction

Rapid and vigorous proliferation of any marine organisms, culminating in predominance within the local population, exerts profound impacts upon both the environment and the other species, thus a comprehensive understanding of these impacts is essential (Blauw et al. 2010, Zohdi and Abbaspour 2019). Extensive attention and research have been focused on blooms of algae and jellyfish to clarify direct and indirect impacts on bacterial communities (Sun et al. 2020, Xia et al. 2020, Peng et al. 2021, Shi et al. 2023). However, our current understanding of these impacts remains insufficient.

The shelled pteropod *Creseis acicula* is a marine pelagic shellfish widely distributed around the world. *Creseis acicula* is primarily found at depths of less than 500 m in both tropical and subtropical waters of the Pacific, Atlantic and Indian Oceans (Albergoni 1975, López-Arellanes et al. 2018, Zeng et al. 2021). *Creseis acicula* blooms have occurred globally, with the highest frequency in the Karwar, Goa and Bengal bays of the Indian Ocean (Krishna-Murthy 1967, Peter and Paulinose 1978, Naomi 1988), the north Pacific waters around Sado Island (Nishimura 1965), the Gulf of Mexico (Hutton 1960) and the Mediterranean Sea (Kokelj et al. 1994, Tunçer et al. 2021)*. Creseis acicula* is commonly the dominant pteropod species in the Yellow Sea, East China Sea and South China Sea (Zhang 1966, Qi et al. 2021). Although widely distributed in the coastal waters of China, its abundance has remained relatively low, usually lower than 0.50 individuals (ind.) m^−3^, and has never bloomed (Qi et al. 2021). However, in the summer of 2020, from mid-June to mid-July, a substantial outbreak of *C. acicula* was observed in southwestern Daya Bay, Guangdong Province, northern South China Sea. This was the first recorded outbreak of *C. acicula* in Chinese waters and also is the highest density (5600 ind. m^−3^) recorded worldwide to date (Qi et al. 2021).

Bacterial communities, either floating in seawater or attached to the surfaces of particulate matter, play crucial roles in the biogeochemical cycles of almost all elements in the ocean ecosystem (Ruiz-Gonzalez et al. 2013, Shi et al. 2023). Additionally, active bacterial exchange occurs with changes to the environmental conditions of two or more habitats, where each habitat favors the proliferation of specific taxa that are typically unrepresentative in the other (De Corte et al. 2014, 2018, Liu et al. 2023). Therefore, we hypothesized that the outbreak of *C. acicula* may exert various impacts on the bacterial communities. For example, the sudden bloom of *C. acicula* provided a substantial number of particles (e.g. the pteropod and fecal pellets) to which some bacterial species could attach, thus favoring their numbers increasing, whereas the abundance of some other bacterial species may decrease due to the filter-feeding of *C. acicula*, which markedly decreases the amount of suspended particulate organic matter (such as diatoms, dinoflagellates and microcrustaceans) to which these bacterial species are attached. However, these hypotheses and assumptions have not yet been conclusively investigated, potentially due to the low frequency of *C. acicula* blooms. The associations and relationships between bacteria and *C. acicula* are far from fully understood.

In the present study, we examined the profiles of three communities of bacteria, that is, the pelagic species floating in the waters (referred to as “free-living bacteria”), the species attached to particles such as the fecal pellets of *C. acicula* (referred as “particle-attached bacteria”) and the bacteria attached to the whole body (soft tissue + shell) and shell of *C. acicula* using a high-throughput sequencing method to gain a better understanding of the potential impacts on other organisms in coastal waters.

## Materials and methods

### Study sites and sampling protocols

**Figure 1.**
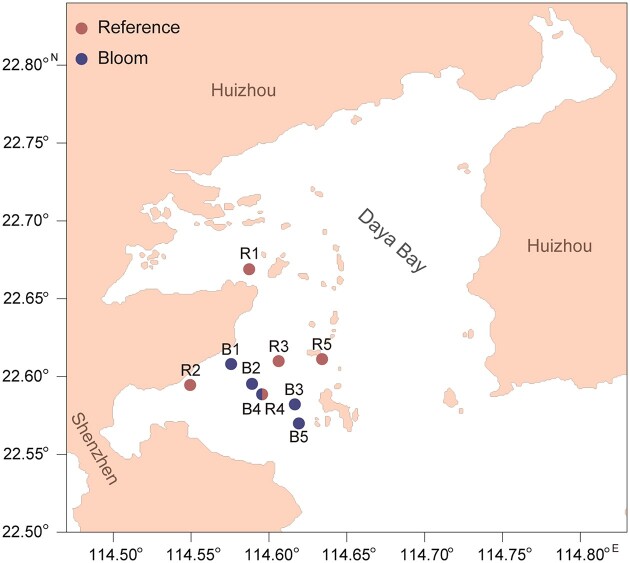
Sampling stations (●) in Daya Bay. Red dots (R1–R5) represent the reference areas and blue dots (B1–B5) represent the *C. acicula* blooming areas. The dot with half-red and half-blue represents a blooming area on July 9 (B4) and a reference area on July 10 (R4), respectively.

This study was carried out in Daya Bay, located in Guangdong Province, southern China, which is a 650 km^2^ semi-enclosed embayment in the northeast of the South China Sea. Its average water depth is 10 (range: 5−20) m and the annual mean air temperature is 22°C. The minimum sea surface temperature occurs in winter (15°C), and the maximum in summer and fall (30°C) (Wang et al. 2006). A bloom of *C. acicula* occurred in southwest Daya Bay from mid-June to mid-July 2020. On 9 and 10 July, triplicate water samples were collected from each station in both blooming areas (n = 15) and adjacent non-blooming (termed as reference) areas (n = 15) (as shown in Fig. 1); 5 L of surface seawater was collected in each station and immediately transported to our seaside laboratory using an ice box for determining the bacterial community structure, picoplankton abundance and the concentrations of NO_3_^−^, NO_2_^−^, NH_4_^+^, PO_4_^3−^, SiO_3_^2−^ and chlorophyll *a*. Additionally, the temperature, salinity, dissolved oxygen (DO) and pH of the surface seawater were measured *in situ* using a multiprobe sonde (Yellow Springs Instrument Company, Inc., Yellow Springs, OH, USA).

In the laboratory, 2.5 L of surface seawater was pre-filtered with a 20-µm bolting cloth to remove debris and larger organisms. Then the number of *C. acicula* individuals retained in the bolting cloth was quantified. For bacterial DNA extraction, 2.5 L of seawater was passed through 3-µm polycarbonate membranes (EMD Millipore Corporation, Billerica, MA, USA), and 1.5 L of the filtrate was passed through 0.2-µm polycarbonate membranes (EMD Millipore Corporation). The particle-attached bacteria (attached to particles with a diameter of 3–20 µm) and free-living bacteria (with a size of 0.2–3 µm) that were captured with the 3- and 0.2-µm membranes, respectively, were frozen in liquid nitrogen and stored at –80°C until DNA extraction (Ortega-Retuerta et al. 2013, Shi et al. 2023).

For the bacteria attached to the whole body (soft tissue + shell) of *C. acicula*, intact *C. acicula* samples (n = 8–10) collected from sites B1, B3 and B4 were transferred to a centrifuge tube containing 5 ml of sterile seawater after washing twice with sterile seawater to remove any free-living bacteria. Samples (sterile seawater containing *C. acicula*) were filtered through 0.2-µm membranes after crushing and oscillating at 1000 r/m for 20 sec with a vortex oscillator (Scientific Industries, Bohemia, NY, USA). The membranes were frozen in liquid nitrogen and stored at –80°C until DNA extraction. Similarly, for the bacteria attached on the shell of *C. acicula*, shells were gently separated from the soft tissues of *C. acicula* (n = 8–10) with a tweezer, then collected and stored as described above.

### Abundance of picoplankton

Triplicate 2-mL seawater samples were treated with 1% glutaraldehyde and stored in liquid nitrogen to determine the abundance of picoplankton (picophytoplankton and free-living bacteria). Picophytoplankton populations of *Prochlorococcus, Synechococcus* and picoeukaryotes were identified by flow cytometry (FACScalibur flow cytometer, Becton Dickinson, San Jose, CA, USA) based on analysis of the parameters SSC, FL2 and FL3 (Olson et al. 1990, Li et al. 2017). Total free-living bacterial abundance (TBA) was determined by staining with SYBR-Green I (Molecular Probes, Eugene, OR, USA) and quantifing cell numbers by flow cytometry. Based on green fluorescence intensity (FL1), the free-living bacteria were segregated into two categories: free-living bacteria with low nucleic acid content (LNA) and free-living bacteria with high nucleic acid content (HNA).

### DNA extraction and high-throughput sequencing

Bacterial DNA was extracted using the FastDNA® Spin Kit for Soil (MP Biomedicals, Inc., Santa Ana, CA, USA), and assessments of its concentration and quality used a NanoDrop™ 2000 UV-vis spectrophotometer (Thermo Fisher Scientific, Waltham, MA, USA) and 1% agarose gel electrophoresis, respectively. The V4–V5 hypervariable regions of the bacterial 16S rRNA gene were amplified using the universal bacterial primers 515F (5′-GTG YCA GCM GCC GCG GTA A-3′) and 926R (5′-CCG YCA ATT YMT TTR AGT TT-3′) with a GeneAmp™ PCR System 9700 (Applied Biosystems, Carlsbad, CA, USA) (Parada et al. 2015). The reaction system and reaction conditions were according to Shi et al. (2023). PCR products were extracted from 2% agarose gel and purified using the AxyPrep DNA Gel Extraction Kit (Axygen Biosciences, Union City, CA, USA) according to the manufacturer's instructions, and were quantified using a Quantus™ Fluorometer (Promega Co., Madison, WI, USA). Purified amplicons were pooled in equimolar amounts and paired-end sequenced on an Illumina MiSeq platform (Illumina Inc., San Diego, CA, USA). The sequences are publicly available at the NCBI Sequence Read Archive (http://www.ncbi.nlm.nih.gov/Traces/sra) under BioProject number PRJNA988167.

### Environmental factors analysis

Samples for chlorophyll *a* were collected by filtering 0.3-L seawater samples to a Whatman GF/F filter (47 mm in diameter), and chlorophyll *a* concentrations were measured with a fluorometer, as described by Parsons et al. (1984), after treating filter membranes with 90% (v/v) acetone for 24 h in the dark. The concentrations of NO_3_^−^, NO_2_^−^, NH_4_^+^, PO_4_^3−^ and SiO_3_^2−^ were measured with a spectrophotometer (Shanghai Precision Science Instrument Co., Ltd., Shanghai, China) in accordance with “Specifications for oceanographic survey” (GB/T 12763.4–2007 2007). The detection limits for NO_3_^−^, NO_2_^−^, NH_4_^+^, PO_4_^3−^ and SiO_3_^2−^ were 0.05, 0.02, 0.03, 0.02 and 0.05 µmol L^−1^, respectively.

### Data analysis

The raw 16S rRNA gene sequencing reads were demultiplexed, quality filtered and merged using fastp version 0.20.0 (Chen et al. 2018) and FLASH version 1.2.7 (Magoč and Salzberg 2011), and the applied criteria were according to Shi et al. (2023). Operational taxonomic units (OTUs) were clustered at a 97% similarity cutoff using UPARSE version 7.1 (Edgar 2013) and rarefied based on the smallest number of sequences (16 405) to standardize uneven sequence depth, and the taxonomy of each OTU representative sequence was analyzed with RDP Classifier version 2.2 against the 16S rRNA gene database (Silva v138.1) with a confidence threshold of 0.7 (Wang et al. 2007).

Statistical analysis was performed using R version 3.6.3 (R Core Team 2018). The bacterial community structure was analyzed using non-metric multidimensional scaling (NMDS) based on Bray-Curtis distances with the R package “vegan”. ANOSIM was used to compare differences both within and between groups with 999 permutations (Oksanen et al. 2019). Normal distribution and homogeneity of variance were analyzed with the R packages “stats” and “car”. A co-occurrence network was established using the R packages “psych” and “igraph” based on the genus level (Csárdi and Nepusz 2006, Revelle 2021). Spearman correlation scores were calculated, and only robust (Spearman's r > 0.7 or r < –0.7) and statistically significant (Benjamini and Hochberg-adjusted *P* < 0.01) correlations were kept in the network (Benjamini et al. 2006, Ma et al. 2016). In the co-occurrence network, bloom-sensitive particle-attached bacteria whose distributions were significant differences (*P* < 0.05) between the blooming and reference areas were identified using indicator species and edgeR analysis and marked in the plot with R software. Differences in the distribution of the bloom-sensitive particle-attached bacteria (with a relative abundance of >0.1%) between the whole body (soft tissue + shell) and shell of *C. acicula* were calculated using STAMP (Parks et al. 2014). Additionally, correlation coefficients between environmental factors and bloom-sensitive particle-attached bacteria, with significant differences in distribution patterns between the whole body (soft tissue + shell) and shell of *C. acicula*, were visualized in heatmaps generated with the R package “pheatmap” (Kolde 2019).

## Results

### Bacterial community structures in the waters between *C. acicula* blooming and reference areas

**Figure 2.**
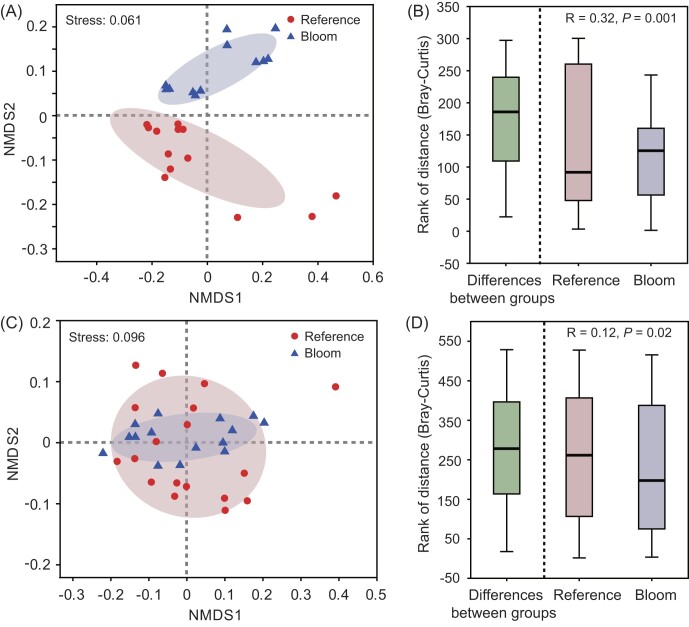
NMDS and distance boxplots of particle-attached bacteria (A, B) and free-living bacteria (C, D) at the genus level.

There was a clear separation of particle-attached bacteria between the blooming and reference areas (Fig.2A). The differences between blooming and reference areas were greater than that within each area (Fig. 2B). The free-living bacterial communities from either blooming or reference areas were mixed (Fig. 2C), thus no obvious differences were observed (Fig. 2D). Therefore, *C. acicula* bloom had a greater impact on particle-attached bacteria than that on free-living bacteria (i.e. the particle-attached bacteria were more sensitive to *C. acicula* bloom).

### Effects of *C. acicula* bloom on co-occurrence patterns of particle-attached bacteria

**Figure 3.**
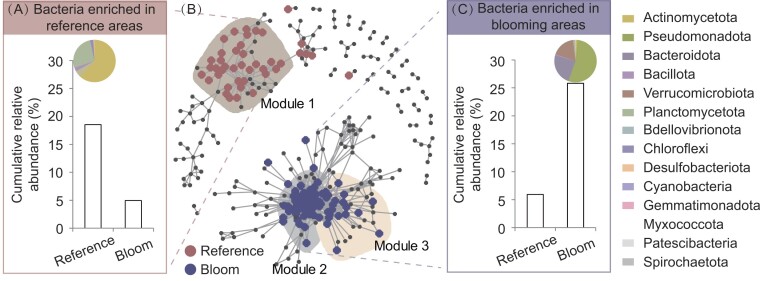
Co-occurrence patterns of *C. acicula* bloom-sensitive particle-attached bacteria. (A) The cumulative relative abundance of bacteria significantly enriched in the reference areas with a pie chart representing the qualitative taxonomic composition at the phylum level. (B) Co-occurrence networks visualizing significant correlations (r > 0.7 or < –0.7, *P* < 0.001) among particle-attached bacteria. Each node in the network represents a bacterial genus and the color represents the association with the bloom (red circles, bacteria significantly enriched in the reference areas; blue circles, bacteria significantly enriched in the blooming areas; gray circles, bacteria insensitive to the bloom). Shading color represents the bacterial distribution modules. (C) The cumulative relative abundance of bacteria significantly enriched in the *C. acicula* blooming areas with a pie chart representing the qualitative taxonomic composition at the phylum levels. A legend for the pie charts (A and C) is shown on the right.

As shown in Fig. 3, there was a clear separation of the bloom-sensitive particle-attached bacteria between the blooming and reference areas. Furthermore, Module 1 mainly consisted of bacteria significantly enriched in the reference areas, with Actinomycetota and Planctomycetota dominating with mean cumulative relative abundances of 62.13% and 26.78%, respectively (Fig. 3A and B). Bacteria in Modules 2 and 3 were significantly enriched in the blooming areas, and these bacteria mainly belonged to Pseudomonadota, Bacteroidota and Verrucomicrobiota [mean cumulative relative abundances of 54%, 23.70% and 17.84%, respectively (Fig. 3B and C)].

### Bacteria attached to the whole body (soft tissue + shell) or shell of *C. acicula*

**Figure 4.**
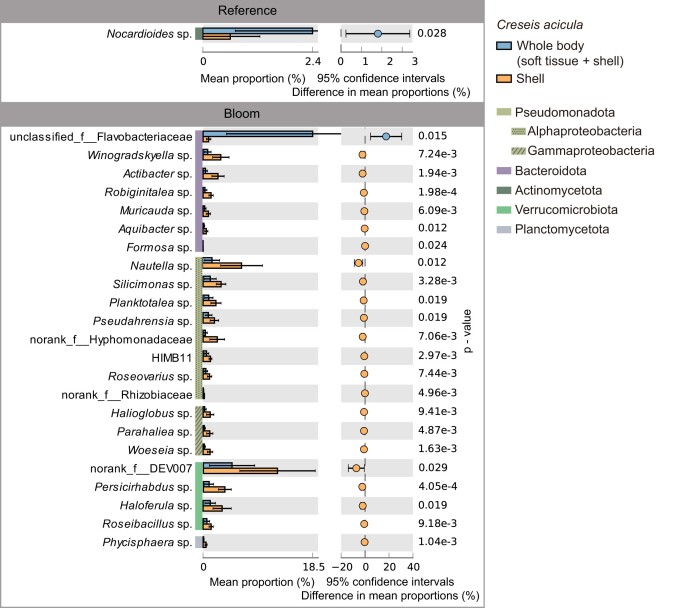
Extended error bar plot of *C. acicula* bloom-sensitive particle-attached bacteria (relative abundance of >0.1%) at the genus level. Genera with significant differences between the whole body (soft tissue + shell) and shell of *C. acicula* (*P* > 0.05) are shown. The error bars represent standard errors of the bacterial proportions.

The distribution differences of the bloom-sensitive particle-attached bacteria on the whole body (soft tissue + shell) and shell of *C. acicula* were determined to clarify potential relationships between these bacteria and *C. acicula* (Fig. 4). Of the bacteria enriched in the reference areas, only *Nocardioides* sp. (Actinomycetota) had a significantly (*P* = 0.028) higher abundance on the whole body than on the shell of *C. acicula* (Fig. 4). Of the bacteria enriched in the blooming areas, the abundance of unclassified Flavobacteriaceae bacteria (Bacteroidota) on the whole body was significantly (*P* = 0.015) higher than that on the shell of *C. acicula*. Conversely, Bacteroidota, Pseudomonadota (Alphaproteobacteria and Gammaproteobacteria), Verrucomicrobiota and Planctomycetota were significantly (*P* < 0.05) more abundant on the shell than on the whole body of *C. acicula*.

### Relationships between bloom-sensitive particle-attached bacteria and environmental factors

**Figure 5.**
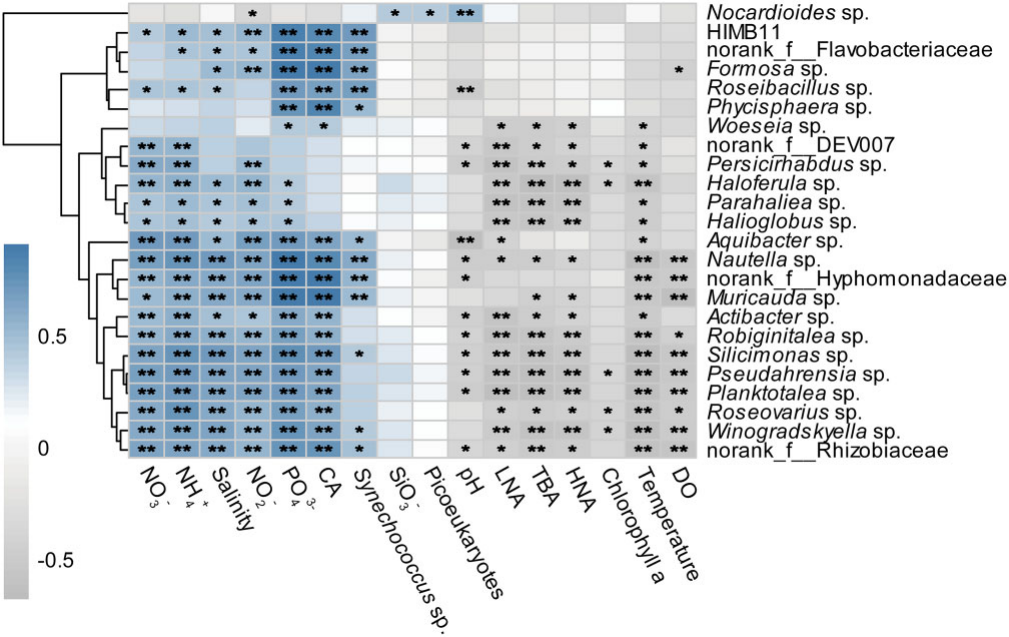
Correlation coefficients between environmental factors and *C. acicula* bloom-sensitive particle-attached bacteria (at the genus level) with significant difference in distribution patterns on the whole body and shell of *C. acicula*. *P < 0.05, **P < 0.01. CA, *Creseis acicula* density; DO, dissolved oxygen; HNA, free-living bacteria with high nucleic acid content; LNA, free-living bacteria with low nucleic acid content; TBA, total free-living bacteria.

For those bloom-sensitive particle-attached bacteria that had significantly different distribution patterns between the whole body and shell of *C. acicula*, their relationships with environmental factors are shown in Fig. 5. The abundance of *Nocardioides* sp. was significantly (*P* < 0.05) and positively correlated with SiO_3_^−^, picoeukaryotes and pH, but was negatively correlated with nutrients (NO_3_^−^, NH_4_^+^, NO_2_^−^ and PO_4_^3−^) concentrations. The abundances of HIMB11, norank_f_Flavobacteriaceae, *Formosa* and *Roseibacillus* spp. were significantly (*P* < 0.01) and positively correlated with *C. acicula* density (CA), *Synechococcus* sp. and PO_4_^3-^ contents. However, *Persicirhabdus, Haloferula, Parahaliea* and *Halioglobus* spp. were significantly (*P* < 0.05) and positively correlated with inorganic nitrogen (NO_3_^−^, NH_4_^+^ and NO_2_^−^), but significantly (*P* < 0.05) and negatively correlated with LNA, TBA, HNA and temperature. *Nautella, Silicimonas* and *Planktotalea* spp. were significantly (*P* < 0.01) and positively correlated with nutrient contents, salinity and CA, but significantly (*P* < 0.01) and negatively correlated with temperature and DO.

## Discussion

Compared with algal blooms (Sun et al. 2020, Xia et al. 2020, Shi et al. 2023), the impacts of *C. acicula* bloom on bacterial communities remain unclear. The results of the present study clearly demonstrated that a *C. acicula* bloom also had direct or indirect influences on bacterial communities. In addition, compared with algae, *C. acicula* had some species-specific impacts. They could migrate in both horizontal and vertical directions (Zeng et al. 2021), instead of passively transporting with current as algae do, and this ability restricted long-term exposure of bacteria to their metabolites (Kornicker 1959, Han et al. 2022). Additionally, unlike the dead algae that floated in the surface waters and decomposed there, the heavy straight-needle shells made the dead *C. acicula* sink rapidly to the seabed where they decomposed, thereby minimizing their impacts on bacteria in the surface waters (Blauw et al. 2010, Zohdi and Abbaspour 2019, Zeng et al. 2021).

Algal blooms appeared to mainly affect the free-living bacteria in the waters instead of those species attached to particles (Shi et al. 2023), whereas *C. acicula* bloom could have various impacts on particle-attached bacteria communities in several ways; for example, on the one hand, *C. acicula* were filter-feeders, they fed on suspended particles including diatoms, dinoflagellates and microcrustaceans, which were substratum for particle-attached bacteria. This process consequently decreased the abundance of these bacteria (Sakthivel and Haridas 1974, Zeng et al. 2021). On the other hand, the fecal pellets released by *C. acicula* provided the substratum for particle-attached bacteria, which had the potential to increase the abundance of these bacterial species. Furthermore, *C. acicula* bloom excreted inorganic nutrients (e.g. NO_3_^−^, NH_4_^+^, PO_4_^3−^ and CO_2_ into water; Table S1) and stimulated the growth of phytoplankton. This also increased the available substratum for particle-attached bacteria. Additionally, bioavailable phosphorus (mainly PO_4_^3−^) could be directly absorbed and utilized by some particle-attached bacterial species such as copepod-attached bacteria, which harbor large numbers of phosphate transport-related genes (De Corte et al. 2018). Therefore, the PO_4_^3−^ excreted by *C. acicula* may favor the growth of copepod-attached bacterial communities.

Particle-attached bacteria with significant differences in distribution patterns between the reference and blooming areas were classified as bloom-sensitive particle-attached bacteria; the distribution of these bacteria into distinct modules of the co-occurrence network was correlated with the presence or absence of *C. acicula* bloom, which indicated that the distributions of these bacteria may relate to *C. acicula*, whose exoskeleton and gut are nutrient- and carbon-enriched microhabitats for colonization by bacteria (Carman and Dobbs 1997, Tang et al. 2010, De Corte et al. 2018). Additionally, these bacteria may originate from the feces or intestines of *C. acicula*, or attach to particles consumed by *C. acicula*. Therefore, it is helpful to analyze the differences in the distribution patterns of bloom-sensitive particle-attached bacteria between the whole body (soft tissue + shell) and shell of *C. acicula*.

For the bloom-sensitive particle-attached bacteria belonging to the phylum Actinomycetota, only the endophytic *Nocardioides* sp. had a significantly higher abundance on the whole body (soft tissue + shell) rather than on the shell of *C. acicula*. Its abundance was positively correlated with the picoeukaryotes abundance, but negatively with the density of *C. acicula*, suggesting that its abundance increases within the *C. acicula* microbiome by “hitchhiking” into the gut on ingested food particles (Benson and Silvester 1993, Anandan et al. 2016). Ultimately, *Nocardioides* sp. accumulated on the whole body of *C. acicula* instead of its shell.

The reasons for the higher abundance of particle-attached bacteria in blooming waters and on *C. acicula* shells could potentially be: (1) as discussed above, the high population of *C. acicula* in blooming areas produced a large number of fecal pellets, which could be the substratum for these bacteria to attach to. Reasonably, some bacteria attach to *C. acicula* shells. For example, in the present study, the abundance of *Halioglobus* and *Haloferula* spp., which were proven to be the dominant bacteria in mussel feces (Griffin et al. 2021), was positive, although the correlation efficiency did not reach a significant level, related to the density of *C. acicula* in the waters; (2) the *C. acicula* shells also served as the substratum for some epizoic diatom *Licmophora* sp., which have specialized mucilage pads and stalks favoring them to adhere on shells (Russell and Norris 1971, Hiromi et al. 1985, Gómez et al. 2018, 2020). Therefore, the abundance of *Licmophora* sp. and bacteria attached to it, such as diatom-associated *Winogradskyella* (Bacteroidota), substantially increased in *C. acicula* bloom areas (Zhang et al. 2023); and (3) some *C. acicula*-associated bacteria, such as members of the family Rhodobacteraceae, significantly proliferated with the *C. acicula* bloom and subsequently adhered to other particles in the environment. Rhodobacteraceae was the dominant family of zooplankton-associated bacteria and could adhere to coral, sponges and microalgae (Roder et al. 2014, De Corte et al. 2018). In the present study, Rhodobacteraceae strains, including *Nautella, Silicimonas, Planktotalea* and *Roseovarius* spp., as well as HIMB11, had accumulated on the shells of *C. acicula* and proliferated in the blooming areas as particle-attached bacteria. Additionally, the abundances of these taxa were significantly and positively correlated with the density of *C. acicula*, which may be because they are contributing to biofilm formation, and thus play a crucial role in the colonization of other microorganisms to gain a competitive advantage for colonization on the shells of *C. acicula* (Amaral-Zettler et al. 2020, Zhao et al. 2023).

Members of the family Flavobacteriaceae, which are able to degrade high molecular organic matter, such as proteins and polysaccharides (Bennke et al. 2016, Lapebie et al. 2019), exhibit commensal or parasitic interactions with zooplankton (Cottrell and Kirchman 2000, Beier and Bertilsson 2013). In this study, the abundance of unclassified Flavobacteriaceae on the whole body of *C. acicula* was significantly greater than that on the shells. Flavobacteriaceae are known to be the dominant species in shellfish guts and feces (Griffin et al. 2021); similarly, they might also be the dominant species in *C. acicula* intestine. Some members of the Flavobacteriaceae family are aerobic or facultative anaerobic species (Lv et al. 2014), and they could proliferate in the gut of *C. acicula*, which is hypoxic but rich in nutrients (De Corte et al. 2018). These bacteria could be released into the seawater along with the excretions of feces.

## Conclusion

The present study showed that *C. acicula* bloom can directly and indirectly influence bacterial communities through physiological processes such as filter feeding, respiration, metabolite release, excretion and decomposition. The influences on particle-attached bacteria are more pronounced than on free-living bacteria. Our findings indicate that the *C. acicula* bloom can lead to various ecological effects; further studies are needed to improve our understanding of its potentially profound effects on the biogeochemical circulation of the marine ecosystem.

## Supplementary Material

fiae034_Supplemental_File
