# Correction to: Responses of attached bacterial communities to blooms of the swimming shelled pteropod *creseis acicula* in Daya Bay, southern China

**DOI:** 10.1093/femsec/fiae108

**Published:** 2024-08-13

## Abstract

Globally abundant open ocean and coastal ocean *Synechococcus* strains preferentially utilize phosphoanhydrides over phosphoesters.

This is a correction to: Rongjun Shi, Tingting Han, Zhanhui Qi, Honghui Huang, Responses of attached bacterial communities to blooms of the swimming shelled pteropod *Creseis acicula* in Daya Bay, southern China, *FEMS Microbiology Ecology*, Volume 100, Issue 6, June 2024, https://doi.org/10.1093/femsec/fiae034

In the originally published version of this manuscript, there was an error in Figure 3. Specifically, there is an extra network diagram on the left side of the figure.

This error has been rectified and the article updated with the correct version of Figure 3.

