# Supplementary material for: Responses of attached bacterial communities to blooms of the swimming shelled pteropod Creseis acicula in Daya Bay, southern China
Source: FEMS Microbiol Ecol. 2024 Mar 23;100(6):fiae034. doi: 10.1093/femsec/fiae034 (PMC11163984; doi:10.1093/femsec/fiae034)
Supplement: fiae034_Supplemental_File [file fiae034_supplemental_file.docx]

Table S1 Significance test in differences of the physical and chemical factors between blooming and reference areas

|  | pH | Salinity | Temperature |  | DO |  | Chl a |  | NO_2_^-^ | NO_3_^-^ | NH_4_^+^ | PO_4_^-^ | SiO_3_^+^ |  | Synechococcus | Microeukaryotes | |  | TAB | HNA | LNA | |  | | CA |
| --- | --- | --- | --- | --- | --- | --- | --- | --- | --- | --- | --- | --- | --- | --- | --- | --- | --- | --- | --- | --- | --- | --- | --- | --- | --- |
|  |  |  | (°C) |  | mg L^-1^ |  | mg m^-3^ |  | (μmol L^-1^) | | | | |  | 10^4^ cells/mL | | 10^6^ events/mL | | | | |  | |  | |
| Reference | 8.10±0.03 | 32.90±0.42 | 28.92±1.33 |  | 6.38±0.19 |  | 0.31±0.17 |  | 0.55±0.26 | 39.46±19.50 | 4.73±0.75 | 0.31±0.19 | 54.10±24.98 |  | 2.04±1.21 | 1.75±1.25 | |  | 2.47±0.42 | 1.88±0.42 | 0.60±0.07 | |  | | 0 |
| Bloom | 8.03±0.04 | 33.27±0.14 | 27.86±0.50 |  | 6.20±0.11 |  | 0.20±0.09 |  | 1.67±0.73 | 107.16±25.35 | 6.08±0.43 | 1.27±0.50 | 56.53±9.86 |  | 3.09±1.17 | 1.46±0.40 | |  | 2.02±0. 42 | 1.53±0.39 | 0.51±0.06 | |  | | 63.74±41.98 |
| t | -3.24 | 1.89 | -1.66 |  | -1.86 |  | -1.26 |  | - | 4.73 | 3.52 | 3.99 | 0.20 |  | 1.40 | -0.50 | |  | -1.60 | -1.40 | -2.20 | |  | | - |
| p | ***1.20E-02*** | 0.10 | 0.13 |  | 0.10 |  | 0.24 |  | 0.10 | ***1.48E-03*** | ***7.89E-03*** | ***3.99E-03*** | 0.84 |  | 0.20 | 0.63 | |  | 0.15 | 0.20 | 0.06 | |  | | ***7.50E-03*** |

Values given are arithmetic mean ± standard deviation (n= 5).

The data in bold and italics indicated the significance of difference (*p* < 0.05).

DO, dissolved oxygen; TBA, total free-living bacteria; HNA, free-living bacteria with high nucleic acid content; LNA, free-living bacteria with low nucleic acid content; CA: *Creseis acicula* density.
